# Integrating ultra-prolonged prone ventilation with mechanical power monitoring in refractory acute respiratory distress syndrome (ARDS): a case report

**DOI:** 10.3389/fmed.2026.1794854

**Published:** 2026-04-13

**Authors:** Ting-Li Zhuang, Shih-Heng Lin, Dong-En Chuang, Han-Lin Hsu, Wan-Wen Huang, Hao Hsieh, Xiao-Yue Chen

**Affiliations:** 1 School of Respiratory Therapy, College of Medicine, Taipei Medical University, Taipei, Taiwan; 2 Division of Respiratory Therapy, Department of Chest Medicine, Taipei Veterans General Hospital, Taipei, Taiwan; 3 Nurse Practitioner, Department of Internal Medicine, Wan Fang Hospital, Taipei Medical University, Taipei, Taiwan; 4 Division of Pulmonary Medicine, Department of Internal Medicine, Wan Fang Hospital, Taipei Medical University, Taipei, Taiwan; 5 Respiratory Therapy, Division of Pulmonary Medicine, Department of Internal Medicine, Wan Fang Hospital, Taipei Medical University, Taipei, Taiwan; 6 Respiratory Therapy, Division of Pulmonary Medicine, Department of Internal Medicine, Shuang Ho Hospital, Taipei Medical University, New Taipei City, Taiwan

**Keywords:** infection, lung injury, lung mechanics, mechanical ventilation, oxygenation

## Abstract

**Introduction:**

Acute respiratory distress syndrome (ARDS) manifests as acute pulmonary inflammation associated with high mortality. While intermittent prone positioning for 12–16 h is an essential intervention in managing severe ARDS, clinical challenges arise when patients exhibit immediate physiological deterioration during supine transitions. Also, the clinical utility of monitoring mechanical power (MP) as a surrogate for lung-protective ventilation during prone ventilation remains unclear.

**Case presentation:**

We report a 55-years-old female patient with influenza A-associated severe ARDS. Despite an initial trial of high-flow nasal cannula (HFNC) therapy, the patient was intubated and initiated on invasive mechanical ventilation for refractory hypoxemia. Conventional 16-h prone positioning cycles were complicated by marked desaturation and MP instability upon transition to the supine position. We implemented a novel strategy of continuous ultra-prolonged prone positioning sustained for 5 consecutive days. The intervention resulted in a gradual improvement in oxygenation and was uniquely accompanied by a steady decline in MP from 18.14 to 12.33 J/min. Stabilization of both oxygenation and ventilatory mechanics subsequently allowed a safe return to the supine position, followed by successful ventilator weaning and extubation.

**Conclusion:**

Ultra-prolonged prone represented a potentially effective salvage strategy for the severe ARDS patient who was intolerant to standard intermittent cycles. Furthermore, longitudinal monitoring of MP may provide crucial physiological guidance, enabling a more individualized and lung-protective approach during prone ventilation.

## 1 Introduction

Acute respiratory distress syndrome (ARDS) is a life-threatening disease that causes high mortality (1), which is characterized by hypoxia and diffuse lung infiltrates, leading to poor long-term outcomes (2, 3). Notably, ARDS associated with influenza A infection is often more severe and exhibits higher mortality, typically ranging from 31% to 44% in intensive care unit (ICU) cohorts (4–6).

Among the available therapeutic strategies for severe ARDS, prone positioning for 16 h combined with a lung-protective strategy using low tidal volumes (6 mL/kg of predicted body weight, PBW) has been found to significantly reduce mortality (7). However, patient responses to prone positioning are highly heterogeneous (8). In addition, premature supination may lead to derecruitment and deoxygenation (9), highlighting the importance of optimizing and individualizing the duration of prone positioning. Also, frequent repositioning may further increase staff workload and resource utilization.

An additional concern is the lack of a standardized set of respiratory parameters for use during prone ventilation. Previous studies have shown that mechanical power (MP) was independently associated with mortality in patients with ARDS (10, 11), suggesting its potential clinical relevance in this population. In the present report, we described a case of severe influenza A-associated ARDS in which profound hypoxemia occurred upon returning to the supine position after the standard 16-h prone ventilation, leading to the implementation of 5 days of ultra-prolonged prone positioning. We also addressed the role of MP monitoring as a dynamic bedside tool to guide lung-protective ventilation throughout prone ventilation. Our case offered a novel perspective on the management of refractory hypoxemia in ARDS.

## 2 Case presentation

### 2.1 Chief complaints and examinations

**FIGURE 1:**
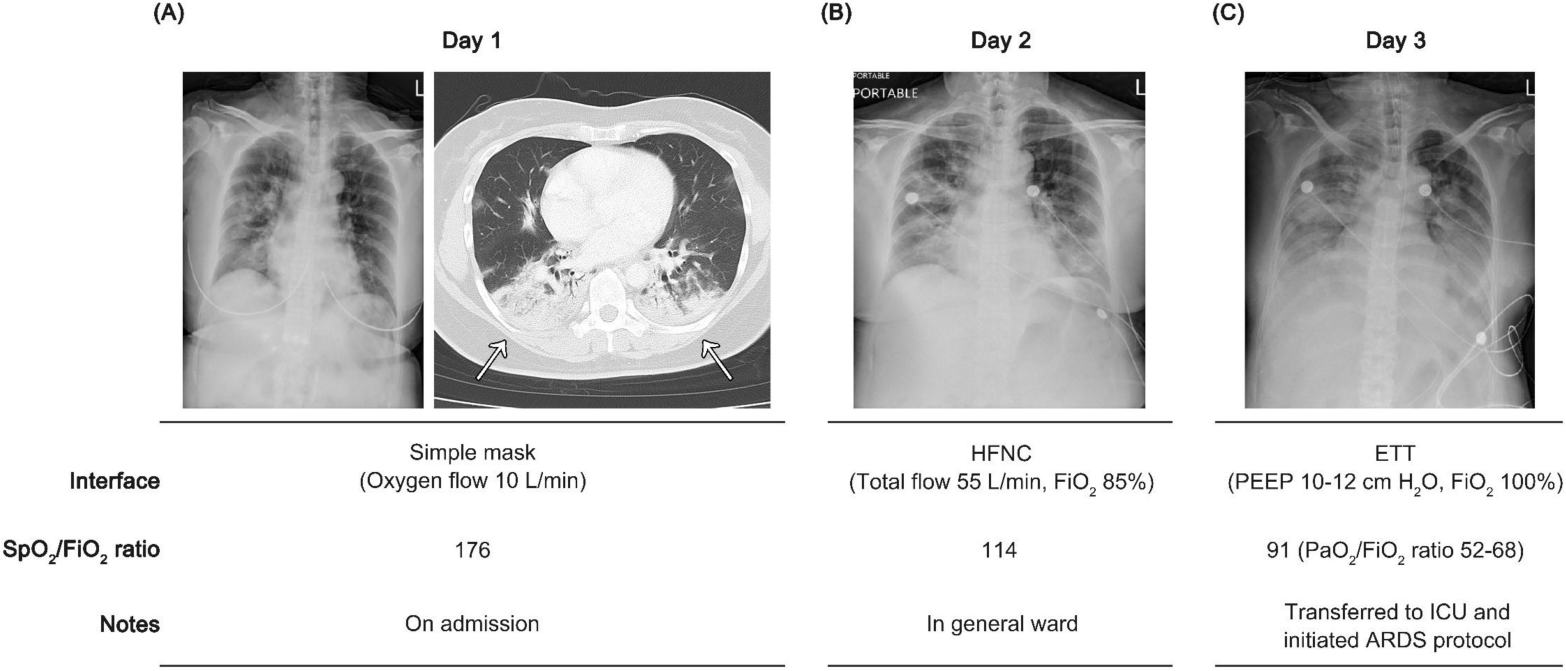
Clinical course and chest images demonstrating progression to acute respiratory distress syndrome (ARDS). **(A)** On admission, the patient received oxygen via a simple mask at 10 L/min due to deoxygenation, with the ratio of oxygen saturation by pulse oximeter to fraction of inspired oxygen (SpO_2_/FiO_2_ ratio) of 176. **(B)** Chest X-ray (CXR) images demonstrated progressive pulmonary infiltrates despite high-flow nasal cannula (HFNC) therapy at 55 L/min and the fraction of inspired oxygen (FiO_2_) 85% on day 2. **(C)** On hospital day 3, the patient was intubated with an endotracheal tube (ETT) and placed on invasive mechanical ventilation, with positive end-expiratory pressure (PEEP) of 10–12 cm H_2_O and FiO_2_ 100%. The SpO_2_/FiO_2_ ratio was 91, and the ratio of partial pressure of arterial oxygen to fraction of inspired oxygen (PaO_2_/FiO_2_ ratio) ranged from 52 to 68, consistent with severe ARDS.

A 55-years-old woman (body mass index: 25.8 kg/m^2^), with a past medical history of treated endometrial hyperplasia and adenomyosis, presented to the emergency department with a 5-days history of high-grade fever (38 °C–39 °C), headache, and cough. Upon admission, the patient had a Glasgow Coma Scale of 15 and an oxygen saturation by pulse oximeter (SpO_2_) of 88% on room air. Due to persistent hypoxemia, supplemental oxygen was administered via a simple face mask at an oxygen flow rate of 10–12 L/min. Auscultation revealed coarse crackles in both lungs. Bilateral white patch infiltrates in the lungs were observed on chest X-ray (CXR) and computed tomography (CT) images (Figure 1A). There were no abnormal findings on electrocardiography. Laboratory data showed slightly elevated blood levels of C-reactive protein (CRP, 26.7 mg/dL), percentage of neutrophil (95%), and aspartate aminotransferase (AST, 46 U/L), while other parameters were within normal limits (Supplementary Table 1).

### 2.2 Diagnosis of ARDS and treatments

Because of severe hypoxemia (the partial pressure of arterial oxygen, PaO_2_, 58 mm Hg) despite oxygen supplementation via a simple mask at 12 L/min, high-flow nasal cannula (HFNC) therapy was initiated on hospital day 2 (Figure 1B) before the family decided on endotracheal intubation, in accordance with ethical considerations. However, hypoxemia persisted, with the ratio of SpO_2_ to fraction of inspired oxygen (SpO_2_/FiO_2_ ratio) at 114 while receiving HFNC at a total flow of 55 L/min and the fraction of inspired oxygen (FiO_2_) 85%, with SpO_2_ ≤ 97%. This met the criteria for non-intubated ARDS (SpO_2_/FiO_2_ ≤ 315) according to the 2023 Berlin definition (12). The respiratory rate-oxygenation (ROX) index was calculated to be 4.39 at 2 h, falling within the range of 2.85–4.87 (13), indicating the need for close and frequent reassessment. However, at 16 h after treatment, the patient experienced desaturation to an SpO_2_ of 85%. HFNC therapy was escalated to a total flow rate of 55 L/min with an FiO_2_ of 95%, achieving an SpO_2_ of 88%–92%. Despite these adjustments, the ROX index further declined to 3.56–3.72, suggesting a high risk of HFNC failure (13, 14). Awake prone positioning was not feasible in our case under HFNC due to patient intolerance related to dyspnea and discomfort, and the patient’s slightly overweight status may have further limited its implementation. Therefore, after confirmation of the patient’s willingness for endotracheal intubation with the family and the chest physician, the patient was intubated with a 7.0-mm internal diameter endotracheal tube secured at 20 cm. Nasopharyngeal swab testing confirmed influenza A infection by polymerase chain reaction (PCR). On the day following intubation, progressive bilateral pulmonary infiltrates without cardiomegaly were observed on CXR images (Figure 1C). Under mechanical ventilation with a PEEP of 10–12 cm H_2_O and FiO_2_ of 100%, the ratio of PaO_2_ to FiO_2_ (PaO_2_/FiO_2_ ratio) was 52–68, and the measured SpO_2_/FiO_2_ ratio was 91, fulfilling the criteria for intubated ARDS. These findings established the diagnosis of severe influenza A-associated ARDS. A lung-protective ventilation strategy was conducted, targeting a tidal volume of 6 mL/kg PBW and a plateau pressure (P_*plat*_) < 30 cm H_2_O. Intermittent neuromuscular blockade by cisatracurium of 0–10 mcg/kg/min was intravenously administered to ensure ventilator synchrony, in accordance with established ARDS management guidelines (15–17). In addition, antiviral therapy with peramivir (300 mg, intravenous infusion, administered twice) was initiated following confirmation of influenza A infection.

### 2.3 Ultra-prolonged prone ventilation

**FIGURE 2:**
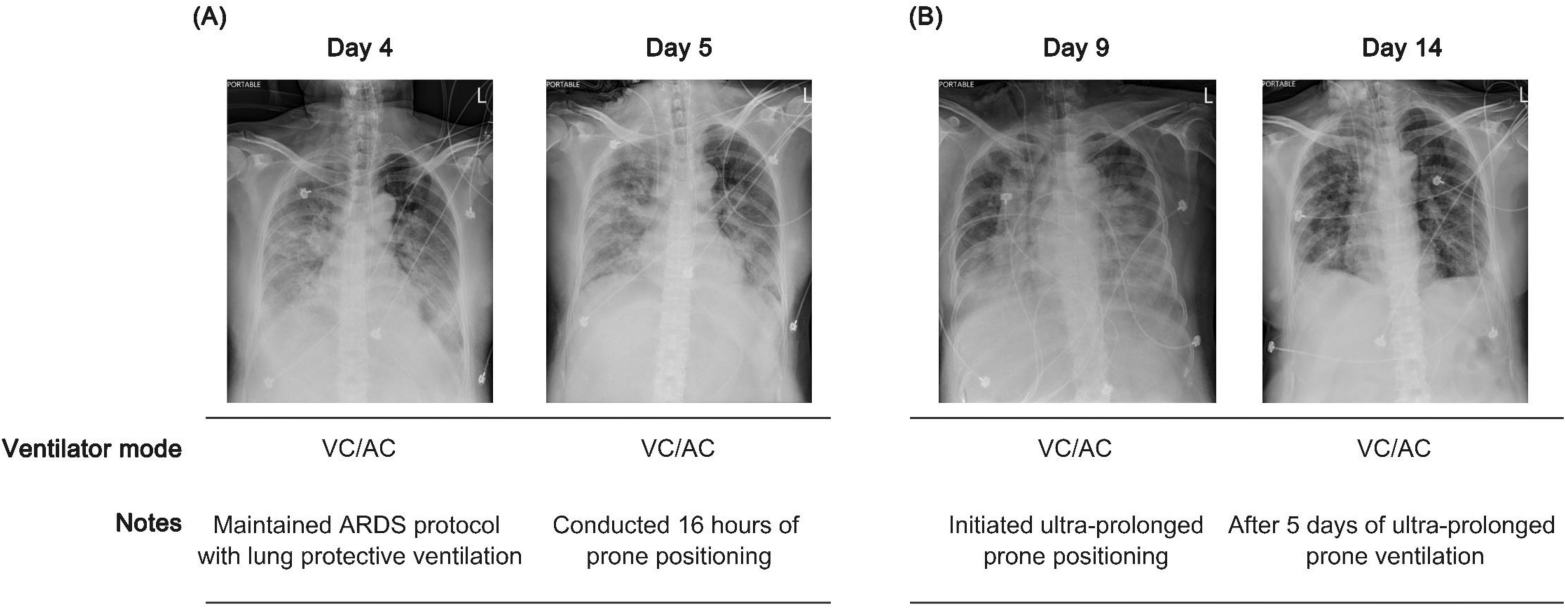
Longitudinal clinical course and radiographic changes during prone ventilation. **(A)** Lung-protective ventilation was applied in volume control/assist-control (VC/AC) mode with 16-h prone sessions. **(B)** Persistent deoxygenation during supine transitions and lack of improvement on chest X-ray (CXR) prompted initiation of ultra-prolonged prone ventilation for 5 consecutive days.

**FIGURE 3:**
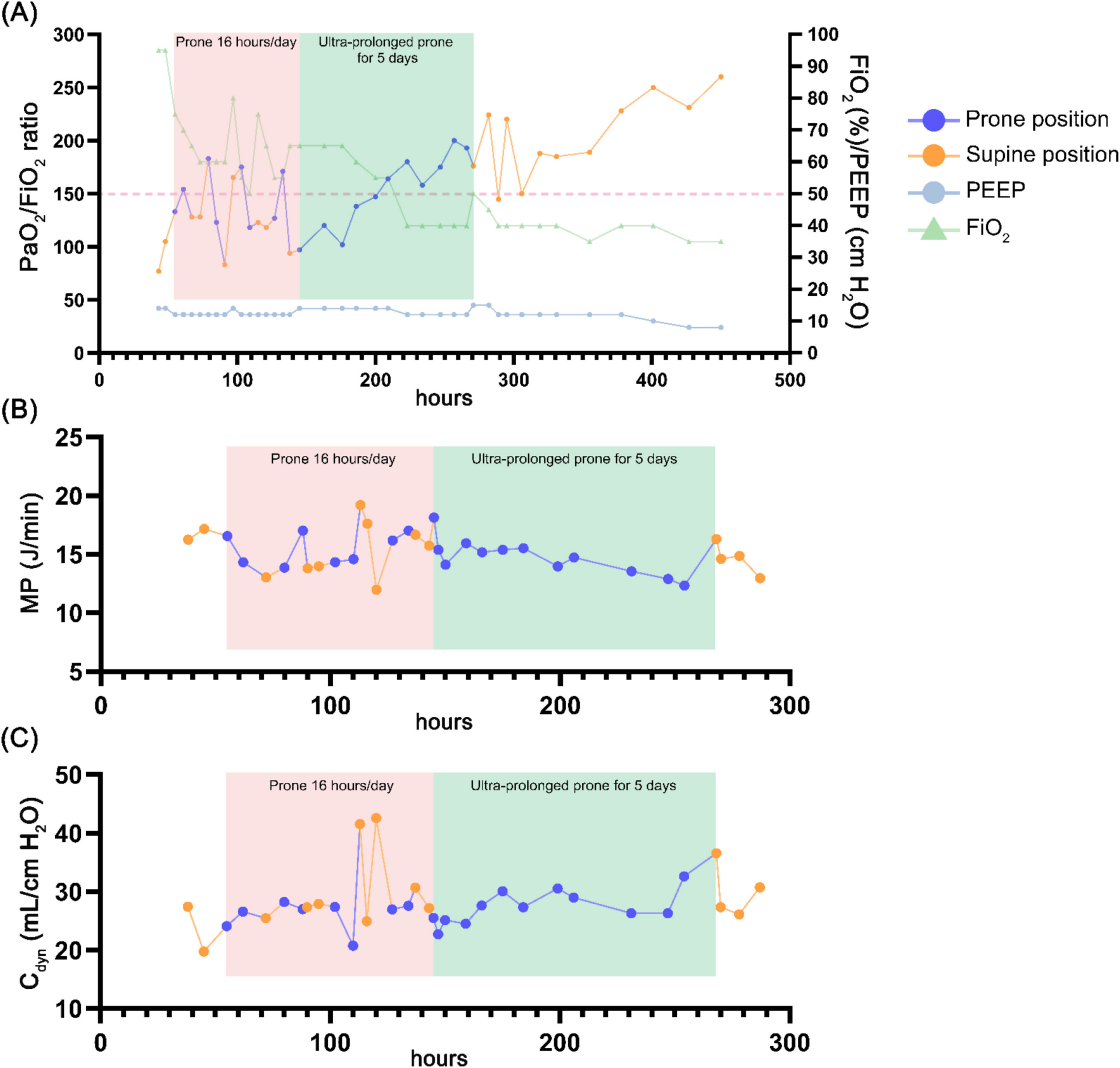
Schematic depiction of the trends in oxygenation and ventilatory mechanics during prone ventilation. **(A)** Trends in the ratio of partial pressure of arterial oxygen to fraction of inspired oxygen (PaO_2_/FiO_2_ ratio), positive end-expiratory pressure (PEEP), fraction of inspired oxygen (FiO_2_), **(B)** mechanical power (MP), and **(C)** dynamic compliance (C_*dyn*_) during standard 16-h prone positioning and ultra-prolonged prone ventilation.

Given that the PaO_2_/FiO_2_ ratio remained ≤150, prone positioning was performed for 16 h on hospital day 5 (Figure 2A). However, upon return to the supine position, the PaO_2_/FiO_2_ ratio rapidly declined, necessitating readjustment of ventilatory support with FiO_2_ > 70%. Due to software limitations on our ventilator (Servo-i; Maquet, Rastatt, Germany), a stepwise lung recruitment maneuver (LRM) was conducted. The initial PEEP was set at 12 cm H_2_O to maintain an SpO_2_ of 88%–95% under baseline settings. PEEP was then increased in increments of 2 cm H_2_O using a stepwise escalation from 12 to 25 cm H_2_O while maintaining a constant driving pressure of 15 cm H_2_O (18, 19). The maximum peak inspiratory pressure was limited to 40 cm H_2_O for 1–2 min (19, 20), after which PEEP was decreased in steps of 2 cm H_2_O. During the decremental phase, SpO_2_ was maintained at 90%–92%, while static compliance (C_*stat*_) values were 26.6, 31.9, and 29.0 mL/cm H_2_O at PEEP levels of 12, 14, and 16 cm H_2_O, respectively. Based on the highest C_*stat*_ and SpO_2_ ≥ 90% without hemodynamic compromise, the LRM was repeated, and the optimal PEEP was set at 14 cm H_2_O. To manage hypoxemia during position transition to supine without repeated LRM, ultra-prolonged prone positioning was conducted for a total of 5 days until oxygenation stabilized (PaO_2_/FiO_2_ > 150) (Figures 2B, 3A). During this period, ventilator settings of FiO_2_ and PEEP were gradually reduced (Supplementary Table 2). Routine nursing care, including side-to-side repositioning every 2 h and head-of-bed elevation at 30–45°, was maintained. Notably, we monitored the MP of the patient, calculated as follows: MP under volume-controlled ventilation (VCV) = 0.098 × respiratory rate × tidal volume × [peak pressure − (plateau pressure − PEEP)/2], according to a previous report (21). We observed that MP fluctuated between 11.98 and 19.22 J/min during the initial 16 h of prone positioning, whereas it showed a steady decline from 18.14 to 12.33 J/min during ultra-prolonged prone positioning (Figure 3B). Following improvement on CXR images with dynamic compliance (C_*dyn*_) increased ≥ 30 mL/cm H_2_O (Figure 3C), the patient was returned to the supine position. She was gradually weaned from the ventilator, extubated on day 22, and successfully discharged.

## 3 Discussion

This report described a case of influenza A-associated ARDS characterized by recurrent oxygen desaturation (PaO_2_/FiO_2_ ratio ≤ 150) during transition from prone to supine positioning after 16 h of prone ventilation. A strategy of ultra-prolonged prone positioning was implemented, extending continuously for 5 days until the PaO_2_/FiO_2_ ratio exceeded 150, with FiO_2_ reduced to 40% (Supplementary Figure 1). During prolonged prone positioning, a steady decrease in MP was observed. The level of C_*dyn*_ increased concurrently with improvement in CXR images. Although mild facial edema occurred during a prolonged prone session, scheduled lateral repositioning and elevation of the head of the bed were maintained, and no other significant complications were identified. Our case demonstrated a novel approach of using uninterrupted, ultra-prolonged prone ventilation to stabilize MP and oxygenation, preventing recurrent desaturation in severe ARDS.

Current therapeutic strategies for ARDS recommend early initiation of prone positioning for 12–16 h per day (15), combined with lung-protective ventilation in patients with severe deoxygenation (PaO_2_/FiO_2_ ≤ 150). However, deterioration in oxygenation, with a PaO_2_/FiO_2_ ratio below 150, may persist, requiring prolonged prone positioning. For example, studies reported that in patients with severe community-acquired pneumonia (CAP)-induced ARDS, prolonged prone ventilation for at least 48–72 h was conducted and resulted in significant improvements in oxygenation (22, 23). In addition, another study demonstrated that maintaining prone ventilation for a median duration of 39 h was safe and increased the PaO_2_/FiO_2_ ratio in patients with coronavirus disease 2019 (COVID-19)-related ARDS (24). Notably, we added to the current knowledge by highlighting that an ultra-extended duration of prone positioning for up to 5 consecutive days was associated with sustained improvement in oxygenation and survival in a case with influenza A-associated ARDS. These observations were consistent with a previous finding showing that extended prone positioning exceeding 24 h improved 30-days and 90-days survival rates (25). However, further studies are needed to elucidate the impact of 5-days consecutive prolonged prone ventilation on mortality. In addition, a prolonged prone strategy may reduce staffing demands associated with frequent patient repositioning (24, 25). In the present case, patient repositioning was performed only 10 times, constituting an approximate 50% reduction compared with the 20 position changes required under a standard prone-ventilation protocol. This reduction may also decrease the risk of unintended endotracheal tube displacement and intravenous catheter dislodgement during position transitions. Also, no severe adverse effects were observed in this case, suggesting the feasibility of the ultra-prolonged prone strategy.

Furthermore, prolonged prone positioning may sustain a more uniform distribution of lung stress and strain (24, 26), reopen refractory dorsal lung collapse (27, 28), and reduce the risk of ventilator-induced lung injury (VILI). In our case, MP and C_*dyn*_ fluctuated during the initial 16-h prone session, potentially due to recurrent overdistension and atelectasis of the lungs. These changes were reflected by marked concomitant fluctuations in the PaO_2_/FiO_2_ ratio. In addition, substantial pressure swings were observed under VCV. This observation aligns with previous findings that peak pressure accounts for most of the increase in MP (29). Moreover, because C_*dyn*_ is calculated based on peak pressure (30), variations in peak pressure inherently influence its magnitude. Therefore, the observed fluctuations in both MP and C_*dyn*_ may reflect the unstable airway pressure during the 16-h prone ventilation. On the other hand, when an ultra-prolonged prone strategy was implemented, a more stable peak pressure was noted, as evidenced by a steady decline in MP from 18.14 to 12.33 J/min and an increase in C_*dyn*_ from 25.49 to 32.60 mL/cm H_2_O. Although reported cut-off values of MP vary between 12 and 18 J/min (31–33), increases exceeding 5 J/min have been associated with higher mortality (31). In contrast, our case showed a steady decrease in MP accompanied by increases in C_*dyn*_ and the PaO_2_/FiO_2_ ratio, suggesting that ultra-prolonged prone positioning may be associated with reduced mortality. This observation warrants further investigation to clarify the clinical implications of ultra-prolonged prone positioning on MP-associated VILI and patient outcomes.

## 4 Conclusion

We presented a case of severe influenza-associated ARDS managed with ultra-prolonged prone positioning under lung-protective ventilation. The intervention was maintained until the PaO_2_/FiO_2_ ratio improved to ≥150 with FiO_2_ 40% and concurrent improvement on CXR images. During ultra-prolonged prone positioning, MP steadily reduced, suggesting a potential mitigation of VILI. In conclusion, ARDS patients who initially receive standard 16-h prone sessions but experience recurrent deoxygenation may benefit from an ultra-prolonged prone strategy. Furthermore, monitoring MP during prone intervention may provide a useful indicator for the risk of VILI and help optimize management in severe ARDS.

## Data Availability

The raw data supporting the conclusions of this article will be made available by the authors, without undue reservation.
