# Supplementary material for: Integrating ultra-prolonged prone ventilation with mechanical power monitoring in refractory acute respiratory distress syndrome (ARDS): a case report
Source: Front Med (Lausanne). 2026 Apr 13;13:1794854. doi: 10.3389/fmed.2026.1794854 (PMC13111545; doi:10.3389/fmed.2026.1794854)
Supplement: Supplementary file 1 [file Data_Sheet_1.doc]

**Integrating Ultra-Prolonged Prone Ventilation with Mechanical Power Monitoring in Refractory Acute Respiratory Distress Syndrome (ARDS): A Case Report**

Ting-Li Zhuang^1,2^ (MSc, RRT), Shih-Heng Lin^1^ (BSc, RRT), Dong-En Chuang^3^ (BSc, NP), Han-Lin Hsu^4^ (MD, MSc), Wan-Wen Huang^5^ (BSc, RRT), Hao Hsieh^6^ (MSc, RRT), Xiao-Yue Chen^1,5*^ (PhD, RRT)

^1^School of Respiratory Therapy, College of Medicine, Taipei Medical University, Taipei, Taiwan

^2^Division of Respiratory Therapy, Department of Chest Medicine, Taipei Veterans General Hospital, Taipei, Taiwan

^3^Nurse Practitioner, Department of Internal Medicine, Taipei Medical University-Wan Fang Hospital, Taipei, Taiwan

^4^Division of Pulmonary Medicine, Department of Internal Medicine, Taipei Medical University-Wan Fang Hospital, Taipei, Taiwan

^5^Respiratory therapy, Division of Pulmonary Medicine, Department of Internal Medicine, Taipei Medical University-Wan Fang Hospital, Taipei, Taiwan

^6^Respiratory Therapy, Division of Pulmonary Medicine, Department of Internal Medicine, Taipei Medical University-Shuang Ho Hospital, New Taipei City, Taiwan

***Corresponding Author**

*Xiao-Yue Chen, PhD, RRT*

Respiratory Therapy Clinical and Translational Research Lab (RT-CTRL), School of Respiratory Therapy, College of Medicine, Taipei Medical University, 250 Wuxing Street, Taipei 110, Taiwan.

Telephone: +886-2-27361661 ext. 3516. E-mail: [xychen@tmu.edu.tw](mailto:xychen@tmu.edu.tw)

**Table S1. Timeline of changes in laboratory parameters.**

| **Hospitalization** | **Day 1** | **Day 2** | **Day 3** | **Day 6** | **Day 8** | **Day 10** | **Day 13** | **Day 15** | **Day 17** | **Day 20** | **Day 22** |
| --- | --- | --- | --- | --- | --- | --- | --- | --- | --- | --- | --- |
| eGFR (mL/min/1.73 m^2^) | 81 | 93 | 105 | 94 | 140 | 172 | 115 | 115 | 109 | 115 | 131 |
| BUN (mg/dL) | N/A | 12 | 13 | 26 | 19 | 19 | 28 | 38 | 43 | 41 | 37 |
| CREA (mg/dL) | 0.79 | 0.70 | 0.63 | 0.69 | 0.49 | 0.41 | 0.58 | 0.58 | 0.61 | 0.58 | 0.52 |
| AST (U/L) | 46 | 76 | 77 | 60 | N/A | 32 | 45 | N/A | 138 | 166 | 71 |
| ALT (U/L) | N/A | 38 | 42 | 84 | N/A | 58 | 46 | N/A | 134 | 219 | 138 |
| PCT (ng/mL) | N/A | 0.57 | 0.47 | N/A | N/A | N/A | N/A | N/A | N/A | N/A | N/A |
| CRP (mg/dL) | 26.7 | 20.8 | 20.9 | 6.4 | N/A | 14.2 | 12.4 | N/A | 13.2 | 11.4 | N/A |
| WBC (x10^3^/μL) | 3.85 | 6.72 | 6.35 | 7.77 | 7.72 | 10.43 | 11.20 | 9.40 | 8.90 | 5.61 | 6.43 |
| Neutrophil (%) | 95.0 | 94.7 | 98.0 | 84.8 | 91.5 | 92.9 | 84.2 | 81.8 | 77.0 | 69.7 | 72.7 |
| Lymphocyte (%) | 3.0 | 1.6 | 1.0 | 4.8 | 1.5 | 1.0 | 4.3 | 6.2 | 8.4 | 10.6 | 14.8 |
| HGB (g/dL) | 12.4 | 11.5 | 11.5 | 10.4 | 10.7 | 10.5 | 9.8 | 10.2 | 9.3 | 8.9 | 8.4 |
| Hct (%) | 37.2 | 34.8 | 34.3 | 31.6 | 33.0 | 31.9 | 30.7 | 30.9 | 28.3 | 27.4 | 26.1 |
| Platelet (x10^3^/μL) | 99 | 140 | 158 | 278 | 303 | 318 | 278 | 255 | 277 | 313 | 393 |
| PT (second) | N/A | 12.7 | N/A | 13.7 | N/A | 14.0 | 14.0 | N/A | 16.1 | 16.5 | N/A |
| aPTT (second) | N/A | 32.5 | N/A | 27.5 | N/A | 32.4 | 32.7 | N/A | 33.9 | 35.2 | N/A |
| INR | N/A | 0.960 | N/A | 1.040 | N/A | 1.070 | 1.060 | N/A | 1.240 | 1.270 | N/A |
| Troponin I (ng/mL) | 0.0326 | N/A | N/A | N/A | N/A | N/A | N/A | N/A | N/A | N/A | N/A |
| NT-proBNP (pg/mL) | N/A | 196 | N/A | N/A | N/A | N/A | N/A | N/A | N/A | N/A | N/A |
| CK (U/L) | 177 | N/A | N/A | N/A | N/A | N/A | N/A | N/A | N/A | N/A | N/A |

Red indicates values above the normal upper limit, whereas blue indicates values below the normal lower limit.

ALT, alanine aminotransferase; aPTT, activated partial thromboplastin time; AST, aspartate aminotransferase; BUN, blood urea nitrogen; CK, creatine kinase; CREA, creatinine; CRP, C-reactive protein; eGFR, estimated glomerular filtration rate; Hct, hematocrit; HGB, hemoglobin; INR, international normalized ratio; N/A, not applicable; NT-proBNP, N-terminal pro-B-type natriuretic peptide; PCT, procalcitonin; PT, prothrombin time; WBC, white blood cell.

**Table S2. Longitudinal ventilator settings from initiation to extubation.**

| **Date** | **Mode** | **Set V_T_ / total V_T_ (mL)** | **Set RR / total RR (bpm)** | **PEEP (cm H₂O)** | **FiO_2_ (%)** | **SpO_2_ (%)** | **SpO_2_/FiO_2_ ratio** | | **Notes** |
| --- | --- | --- | --- | --- | --- | --- | --- | --- | --- |
| **Day 2** | HFNC | N/A | /26 | N/A | 85 | 97 | 114 | |  |
| **Day 3 a.m.** | PC/AC | /459 | 18/18 | 10 | 100 | 91 | 91 | | Intubation |
| **Day 3 p.m.** | VC/AC | 320/318 | 18/35 | 12 | 100 | 98 | 98 | | Followed ARDS protocol  (V_T_ 6 mL/kg PBW) |
| **Day 4** | VC/AC | 320/336 | 16/24 | 14 | 90 | 93 | 103.3 |  | |
| **Day 5** | VC/AC | 320/347 | 22/22 | 12 | 75 | 98 | 130.7 | Prone 16 hours/day | |
| **Day 6 a.m.** | VC/AC | 320/318 | 20/20 | 12 | 60 | 95 | 158.3 | Supine | |
| **Day 6 p.m.** | VC/AC | 320/318 | 20/20 | 12 | 60 | 98 | 163.3 | Prone 16 hours/day | |
| **Day 7** | VC/AC | 320/319 | 20/20 | 12 | 80 | 91-93 | 116.3 | Supine | |
| **Day 9** | VC/AC | 320/327 | 20/20 | 14 | 65 | 96 | 147.7 | Prone for 5 days (day 1) | |
| **Day 10** | VC/AC | 320/341 | 20/20 | 14 | 65 | 94 | 144.6 | Prone for 5 days (day 2) | |
| **Day 11** | VC/AC | 320/342 | 20/20 | 14 | 60 | 96 | 160 | Prone for 5 days (day 3) | |
| **Day 12** | VC/AC | 320/321 | 20/20 | 14 | 40 | 94 | 235 | Prone for 5 days (day 4) | |
| **Day 14 a.m.** | VC/AC | 320/324 | 20/22 | 12 | 40 | 99 | 247.5 | Prone for 5 days (day 5) | |
| **Day 14 p.m.** | VC/AC | 320/313-320 | 20/21-23 | 12 | 40 | N/A | N/A | Supine | |
| **Day 15** | PC/AC | /541 | 12/4 | 12 | 40 | 97 | 242.5 |  | |
| **Day 19** | PC/SIMV+PS | /500-747 | 8/15 | 10 | 40 | 94 | 235 | Started the weaning process | |
| **Day 20** | PSV | /623 | /13 | 10 | 35 | 93 | 265.7 |  | |
| **Day 22** | HFNC | N/A | /17 | N/A | 42 | 97 | 231 | Extubation | |

ARDS, acute respiratory distress syndrome; FiO_2_, fraction of inspired oxygen; HFNC, high-flow nasal cannula; N/A, not applicable; PBW, predicted body weight; PC/AC, pressure control/assist-control; PC/SIMV+PS, pressure control/synchronized intermittent mandatory ventilation with pressure support; PEEP, positive end-expiratory pressure; PSV, pressure support ventilation; RR, respiratory rate; SpO_2_, oxygen saturation by pulse oximeter; SpO_2_/FiO_2_ ratio: the ratio of SpO2 to fraction of inspired oxygen; V_T_, tidal volume; VC/AC, volume control/assist-control.

**Figure S1.** **Summary of clinical course and management in a case with refractory ARDS.**The patient developed hypoxemic respiratory failure while receiving high-flow nasal cannula therapy (HFNC, 55 L/min, FiO_2_ 85%), with a low oxygen saturation by pulse oximeter to fraction of inspired oxygen ratio (SpO_2_/FiO_2_ 114), and underwent endotracheal tube (ETT) intubation. Lung-protective ventilation with a tidal volume of 6 mL/kg predicted body weight (PBW) and a plateau pressure (P_plat_) <30 cm H_2_O was maintained, along with 16-hour daily prone positioning. Due to recurrent oxygen desaturation upon transition to the supine position, an ultra-prolonged prone positioning strategy was implemented for 5 consecutive days. Oxygenation subsequently improved (PaO_2_/FiO_2_ >150), with FiO_2_ reduced to 40% and concurrent improvement in pulmonary infiltrates on chest X-ray (CXR) images. The patient was successfully weaned from mechanical ventilation and extubated.

**
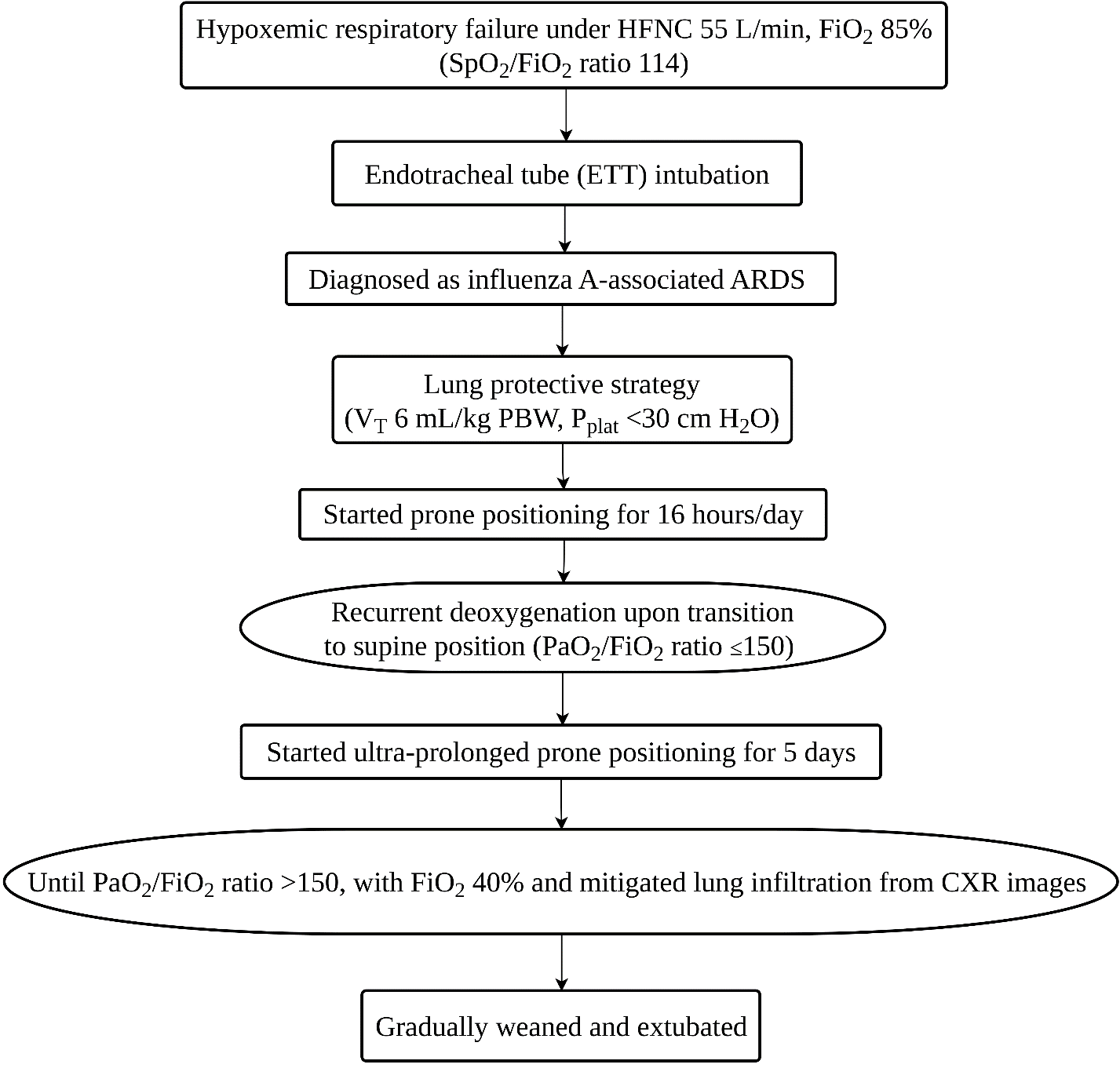
**

**Figure S1.**
